# Gallstone Ileus Decades after Cholecystectomy and Pylorus-Preserving Whipples

**DOI:** 10.1155/2020/8866254

**Published:** 2020-11-27

**Authors:** Miho Mugino, Takako Eva Yabe, Bruce Ashford

**Affiliations:** ^1^ISLHD Division of Surgery, Level 2 Block B Wollongong Hospital, Loftus Street Wollongong NSW 2500, Australia; ^2^Division of Surgery, Wollongong Hospital, Illawarra Shoalhaven Local Health District, Wollongong NSW, Australia

## Abstract

We report a case of small bowel obstruction due to gallstone ileus found in a patient with previous pancreaticoduodenectomy (Whipple procedure). Investigation by computed tomography of the abdomen showed a transition point in the midjejunum due to a radioopaque intraluminal mass. Following resuscitation, the patient underwent laparotomy to remove the offending mass from the midjejunum. Subsequent stone analysis confirmed a cholesterol-rich gallstone. This is thus the first description of gallstone ileus following Whipple procedure. The rarity of this presentation and a literature review is presented.

## 1. Introduction

Gallstone ileus, defined as a mechanical small or large bowel obstruction secondary to impaction of cholelithiasis, has been reported to account for 0.1-5% of bowel obstructions [1]. The most common pathogenesis of gallstone ileus is a biliary fistula from the gallbladder to the duodenum, with the most common site of obstruction being at the ileum (~60.5%) [2].

Gallstone ileus in postcholecystectomy patients is extremely rare [3]. Case reports of previously spilt gallstone passing into the small bowel from the duodenal diverticulum [4] and common bile duct have been described after cholecystectomy [5]. Gallstone ileus after cholecystectomy at the biliary intestinal limb of gastrointestinal anastomosis has been described in the literature [6].

We report a case of a 68-year-old patient with neurofibromatosis type I who presented with gallstone ileus20 years after cholecystectomy and 14 years after pylorus-preserving pancreaticoduodenectomy (Whipple procedure) for ampullary somatostatinoma.

## 2. Case

In April 2020, a 68-year-old female presented to the emergency department with a three-day history of high fevers, epigastric pain, and vomiting. Her medical history was significant for obesity (body mass index 33 kg/m^2^), type I neurofibromatosis, polycystic kidney disease, and portal vein thrombosis with chronic portal hypertension and grade I oesophageal varices. She had total abdominal hysterectomy for dysfunctional uterine bleeding and cholecystectomy for cholelithiasis in 2000. In 2006, she presented with cholangitis and was found to have an ampullary somatostatinoma, for which she required pylorus-preserving pancreatoduodenectomy with single-loop Billroth II gastrojejunostomy reconstruction. The dunked pancreatic anastomosis was created with PDS; the choledochojejunostomy and gastrojejunal anastomosis was created with a single layer running absorbable monofilament suture (Monocryl). She developed exocrine and endocrine pancreatic insufficiency as a consequence.

On initial examination, she was febrile (39.0C), tachycardic (HR 100) with systolic blood pressure of 120. Her abdomen was distended with generalised abdominal tenderness on examination, worst in the epigastrium and right upper quadrant without peritonism.

**Figure 1:**
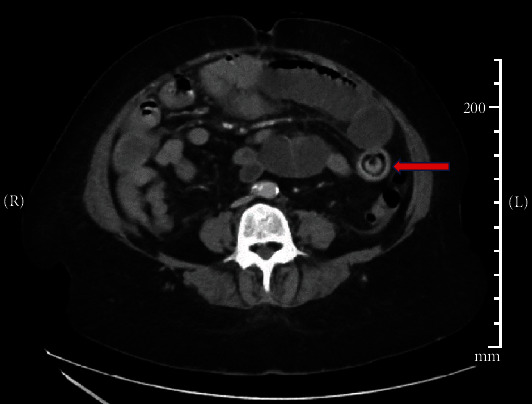
Axial CT demonstrating intraluminal mass causing SBO.

Blood tests were consistent with an acute inflammatory process: elevated white blood cell count (24 × 10^9^/L) and elevated lactate (3.0 mmol/L) which improved after fluid resuscitation. Bilirubin was elevated on admission (40 *μ*mol/L) with mildly elevated gamma-glutamyl transferase (183 U/L) but otherwise normal liver enzymes. Lipase was low at 7 U/L, consistent with pancreatic insufficiency. Abdominal CT (Figure 1 revealed small bowel obstruction (SBO) with a discrete intraluminal mass described as “target-like” acting as a transition point distal to the gastrojejunal anastomosis. She did not have any history of foreign material ingestion, and gallstone ileus was thought to be unlikely given the absence of the gallbladder. As CT report suggested other soft tissue masses in keeping with history of neurofibroma, we suspected this mass to be a neurofibromatosis-related lesion.

**Figure 2:**
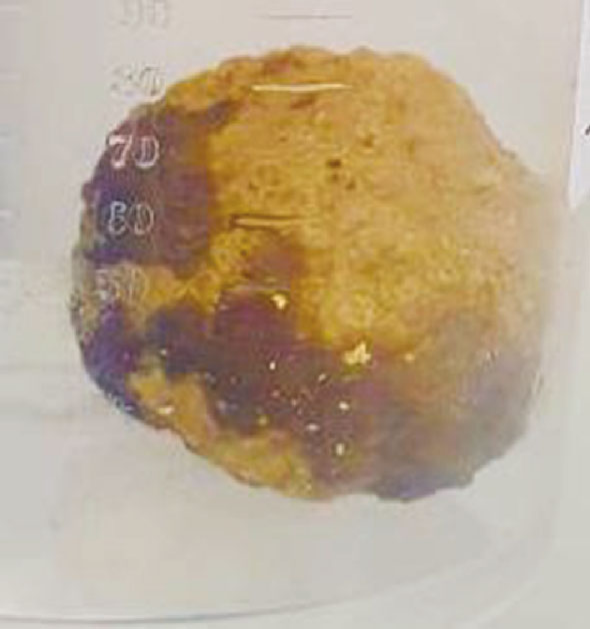
Stone extracted from the small bowel (1.8 cm).

Due to her high fevers and mild cough, she was tested for COVID-19 initially, which was reported negative. Exploratory laparotomy was performed within the first 24 hours of admission. A large amount of intraperitoneal free fluid was encountered upon entry. A mobile rock-hard mass was found in the midjejunum as the cause of small bowel obstruction (Figure 2). The mass was removed by enterotomy which was repaired primarily with 3-0 Polydioxanone (Ethicon, Johnson & Johnson). Laboratory analysis identified this mass as a biliary cholesterol stone. She was started on total parenteral nutrition (TPN) the day after laparotomy for anticipated prolonged ileus and to prevent starvation ketoacidosis. Her enteral nutrition was reintroduced on postoperative day 6, and TPN was ceased. She recovered from the surgery well and was discharged to a rehabilitation facility 16 days after surgery.

## 3. Discussion

This is the first case report of gallstone ileus in a patient who had a pancreaticoduodenectomy. We hypothesise that a biliary stone was formed over fourteen years in the afferent jejunal loop of the gastrojejunostomy and migrated to the efferent limb to cause SBO. We believe that the stasis of bile and cholesterol-rich fluid in the afferent limb precipitated the intraluminal stone formation. This patient might have been suffering from subacute afferent loop syndrome for a long time, which might account for her history of unexplained intermittent upper abdominal pain. Afferent loop syndrome is a rare complication after gastrojejunostomy reconstruction (Billroth II, loop gastrojejunostomy, Roux-en-Y reconstruction) with incidence between 0.3 and 1% [7].

Gallstone ileus in the absence of the gallbladder is extremely rare. Only a handful of case reports have been published between 1939 and 2015. Lee et al. in 2015 described a patient who had “curative resection” of extrahepatic bile duct cancer with Roux-en-Y hepaticojejunostomy. Their patient presented with obstructive jaundice and afferent loop syndrome with CT showing radiopaque stone (2.5 × 1.7 cm) within the hepaticojejunostomy limb. This patient's symptoms spontaneously improved without surgical intervention [6]. Unfortunately there were no operative details or stone analysis provided to demonstrate if this was truly a case of gallstone ileus.

In the setting of the upper gastrointestinal resection, alimentary continuity is maintained by the anastomosing jejunum to the transected ends of the biliopancreatic and gastric lumen. The afferent loop carries high concentration of pancreatic and biliary secretion to the stomach. When a flow of such digestive secretions is compromised, it may cause an increase in back pressure to the biliary system resulting in ascending cholangitis or pancreatitis [8]. This patient presented initially with high fevers, right upper quadrant pain, and obstructive liver function tests (LFT) which could have been a cholangitic manifestation of the “afferent loop syndrome,” especially as bilirubin normalised immediately postoperatively.

The mechanism of gallstone lithogenesis is described by Small's triangle. It demonstrates how the imbalance in the ratio of bile salt, cholesterol, and lecithin causes micellar disequilibrium of soluble bile. This derangement leads to cholesterol stones [9]. Studies on the bile composition pre- and postcholecystectomy indicated that bile composition approaches the micellar zone of cholesterol solubilisation after cholecystectomy, possibly due to the continuous hepatic flow of bile without stasis [10]. According to these findings, gallstone lithogenesis should be less favourable in the post resection state. It can be hypothesised that the afferent limb acted as a “neogallbladder” to store and concentrate the biliary-cholesterol complex.

Furthermore, this patient was predisposed to impaired gastrointestinal motility. Gastric and intestinal motility is maintained by “migrating motor complex” under neural and hormonal control [11]. The duodenum is an important secretory organ for migrating motor complex through production of peptide hormones: notably motilin, gastrin, cholecystokinin, and pancreatic polypeptide [12]. During the Whipple procedure, the duodenum segment is resected and visceral autonomic output is lost due to extensive lymphadenectomy around the celiac axis. The loss of autonomic innervation compounded by loss of prokinetic hormones from the duodenum could have caused stasis in the afferent limb.

Our patient had multiple interesting medical comorbidities. There is no clear causative relationship found between neurofibromatosis type I and gallstone disease. In this patient, the histopathology of the gallbladder in 2000 showed chronic cholecystitis with no malignancy. She developed type I diabetes mellitus (DM) after pancreatectomy. Insulin resistance found in type II DM leads to general increase in plasma insulin level which predisposes cholesterol gallstone formation by increased secretion of cholesterol in the bile [13], as well as autonomic neuropathy that impairs sensitivity to cholecystokinin and the gallbladder's emptying function [14] and impaired sensitivity to cholecystokinin. Direct association between type I DM and gallstone disease, however, remains uncertain with studies showing conflicting results [15].

## 4. Conclusion

We present an unusual presentation of gallstone ileus. Each lithogenic factor discussed in this report appears to have contributed to intraluminal gallstone formation and led to gallstone ileus. It is important to revisit the basic science when we are faced with rare and unusual cases such as this.
